# A critical review of COVID-19 course and vaccination in dermatology patients on immunomodulatory/biologic therapy: recommendations should not differ between non-pregnant and pregnant individuals

**DOI:** 10.3389/fmed.2023.1121025

**Published:** 2023-06-02

**Authors:** Tassahil Messas, Rachel K. Lim, Laura Burns, Sara Yumeen, George Kroumpouzos

**Affiliations:** ^1^Department of Dermatology, University Hospital Centre, University of Constantine III, Constantine, Algeria; ^2^Alpert Medical School, Brown University, Providence, RI, United States; ^3^Department of Dermatology, Alpert Medical School, Brown University, Providence, RI, United States; ^4^GK Dermatology, PC, South Weymouth, MA, United States

**Keywords:** COVID-19, immunomodulator, biologic, COVID-19 vaccine, risks, dermatology, pregnancy, immunosuppressive therapy

## Abstract

COVID-19 can have detrimental effects on immunosuppressed patients. Here, we evaluate the evidence regarding continuing immunomodulatory/biologic (IMBI) therapy in pregnant dermatology patients during the COVID-19 pandemic. Also, we discuss the risks of COVID-19 vaccination in pregnant dermatology patients on IMBI therapy. As indicated in this review, regarding continuing IMBI therapy in pregnant dermatology patients during the pandemic, there is no compelling reason for treating them differently than non-pregnant. The body of evidence indicates that mRNA COVID-19 vaccines are safe during pregnancy. Studies on rheumatology patients, a group that overlaps significantly with the dermatology group, provided essential findings. IMBI in a non-pregnant rheumatology patient was not associated with COVID-19 mortality (except for rituximab), and vaccination of the rheumatology patient during pregnancy improved the obstetric outcomes compared to the unvaccinated patient. Based on this data, it can be stated that after weighing the benefit–risk profile of the available COVID-19 vaccines, the recommendation for the pregnant dermatology patient speaks in favor of the COVID-19 vaccination. COVID-19 vaccine recommendations in pregnant dermatology patients on IMBI should not differ from those for their non-pregnant counterparts.

## 1. Introduction

Since the beginning of the COVID-19 pandemic there have been increasing concerns about the effects of the infection on vulnerable groups, such as patients on immunomodulatory/biologic (IMBI) therapy (immunomodulatory drugs are categorized into immunostimulators or immunosuppressives, and biologics are drugs with a targeted action on the immune system) and pregnant persons. Similar concerns were raised about the safety and efficacy of COVID-19 vaccination in these groups. Such individuals have been traditionally excluded from clinical trials, which made formulating recommendations challenging.

The modulations of the maternal immune system in pregnancy may affect the response to viral infections such as COVID-19 (1). The altered inflammatory response to viruses during pregnancy is thought to be mediated, at least in part, by the following: a shift toward the T helper 2 (Th2) phenotype over Th1 (2); a decrease in circulating natural killer cells (3); a decrease in circulating plasmacytoid dendritic cells (4) that are key for type 1 interferon production against viruses; and an increase in circulating progesterone levels (5). Progesterone can enhance lung repair of damage induced by the influenza virus (6). However, in mouse models, progesterone challenge resulted in a decrease in virus-specific antibody levels and virus-specific CD8+ T cells (7) and alterations in the innate immune system, including the pattern recognition receptors Toll-like receptors (TLRs) during pregnancy (8). COVID-19 infection causes pyroptosis of host cells and release of endogenous molecules created upon tissue injury called damage-associated molecular patterns (DAMPs), which can be TLR ligands and further enhance inflammation. These modulations in the maternal immune system have consequences for the clinical trajectory of COVID-19 and for the treatment and prevention of COVID-19 in pregnancy. However, it remains to be determined whether these adaptations result in a higher susceptibility and/or morbidity, or are in fact, protective against COVID-19 (2).

Here we discuss the effects of COVID-19 on maternal and fetal/neonatal outcomes and evaluate the evidence regarding the continuation of IMBI therapy for dermatologic conditions in pregnant persons diagnosed with COVID-19. We also discuss the evidence relevant to the risks of COVID-19 vaccination in pregnant patients on IMBI therapy for dermatologic conditions. Furthermore, we compare such evidence to that of non-pregnant patients on IMBI therapy.

The scarcity of data and heterogeneity of articles precluded a systematic review. Our literature search focused on articles with information on COVID-19 in pregnant women on IMBI. We searched PubMed and Google Scholar databases since inception using terms including “COVID-19 OR SARS-CoV-2,” “COVID-19 vaccine OR COVID-19 vaccination,” “risks,” “complications OR adverse events,” “immunosuppressive,” “immunomodulator,” “biologic,” “dermatology OR dermatologic,” “skin disease” “pregnancy OR gestation.” Expert opinions and medical society guidance were considered. The reference lists of selected articles were reviewed. The discussion here is limited to IMBI therapies that may be used in pregnancy, including systemic corticosteroids, cyclosporin, tumor necrosis factor alpha inhibitors (TNF*α*I) (e.g., adalimumab, certolizumab pegol, etanercept), interleukin (IL)-17 (e.g., secukinumab), IL-4/13 inhibitors (dupilumab), and anti-IL-12/23 (ustekinumab) (9). Supplementary Tables S1 and S2 detail the pregnancy labeling of these drugs. IMBI that may not be prescribed in pregnancy, such as methotrexate, azathioprine, mycophenolate mofetil, and rituximab is not included.

## 2. COVID-19 effects on maternal course, pregnancy, and neonatal outcomes

### 2.1. Maternal course

The SARS-CoV-2 infection affects 3–20% of pregnant women presenting for labor and delivery (10, 11). In a systematic review, 6–8% of pregnant people universally screened for COVID-19 tested positive, 54–77% of these individuals were asymptomatic, and pregnant people were more likely to be asymptomatic than nonpregnant people of reproductive age with COVID-19 (12). Pregnant patients with asymptomatic infection are at higher risk only for maternal morbidity and preeclampsia (13). Symptoms resemble those in non-pregnant individuals (14, 15). The presence of symptoms increases the risk of severe infection and maternal complications (12, 13). Pregnant women do not appear more likely to contract the infection than the general population. However, pregnancy appears to worsen the clinical course of COVID-19 as indicated by increased risks for intensive care unit admission, need for mechanical ventilation and ventilatory support, and death (12, 14, 16, 17).

Data from 463 US hospitals demonstrated that maternal mortality during childbirth hospitalization increased compared to pre-pandemic (18). Population-based cohort studies have reported increased risks for severe maternal morbidity or mortality in patients with COVID-19 (19, 20). A meta-analysis of observational studies of SARS-CoV-2 infection during pregnancy noted that patients with COVID-19 had a 62% increased risk of developing preeclampsia (21). However, some laboratory findings of SARS-CoV-2 infection can be similar to those seen in severe preeclampsia and HELLP syndrome. Obesity, age greater than 35, hypertension, diabetes, multiple comorbidities, and being unvaccinated are risk factors for serious infection and death in pregnancy (22, 23). The SARS-CoV-2 Delta variant was associated with higher rates of severe maternal morbidity events compared with other strains (24).

### 2.2. Pregnancy and neonatal outcomes

Maternal infection following 20 weeks of gestation raises the risk of negative obstetric outcomes, and infection following 26 weeks raises the risk of unfavorable neonatal outcomes (25). Thus, early vaccination to decrease the risk of contracting SARS-CoV-2 infection is advised. Pregnant women should be given two doses of a mRNA vaccine for more robust maternal and fetal antibody responses (26). The body of evidence suggests that the risks of miscarriage and congenital anomalies are not increased above baseline (27–30). Preterm and cesarean birth rates have been increased in many studies (12, 19, 31, 32). In cohort studies, the increased risk appears to be limited to patients with severe or critical disease in late pregnancy (31, 33, 34), and underlying comorbidities may also play a role. Infection with the Delta variant during pregnancy may be associated with a higher risk of placental dysfunction and fetal compromise than previous variants (35).

Still, more than 95% of newborns of affected mothers are uninfected and in good health (36, 37). Affected newborns tend to present a benign disease course despite the increased need for mechanical ventilation (38). The risk of developing severe COVID-19 appears to be higher in preterm infants and neonates with comorbidities (38). Neonatal morbidity (e.g., need for mechanical ventilation) has been predominantly related to prematurity and adverse uterine environments resulting from critical maternal COVID-19 (12). A systematic review reported that the incidence of neonatal deaths was equivalent between SARS-CoV-2-positive females compared with seronegative counterparts admitted to labor and delivery (39). A US study of over 8,000 stillbirths suggested that pregnant females with COVID-19 have a higher risk of stillbirth than pregnant females without COVID-19 (40). However, data on stillbirths are affected by several confounders and remain inconclusive (36). Some newborns of affected mothers developed a transient rash (41). One reported infant had a diffuse, maculopapular rash that disappeared in one day, while another infant had a diffuse, miliaria-like eruption that resolved in 10 days without therapy (42).

The average rate of SARS-CoV-2 congenital infection (i.e., intrauterine transmission) is less than 2% of maternal infections (43). Shah and colleagues’ criteria suggest that congenital infection be identified by a polymerase chain reaction in umbilical cord blood, neonatal blood, or amniotic fluid obtained during the first 12 h of birth (44). A systematic review that used Shah and *colleagues’* criteria on 47 studies revealed that vertical transmission was confirmed in 0.3%, probable in 0.5%, and possible in 1.8% (45). Individuals with COVID-19 mostly have transient, low-viremia rates (46), which explains why *in-utero* transmission is uncommon (47). However, *in-utero* transmission has been documented (48). The angiotensin-converting enzyme 2 receptors and serine protease TMPRSS2 required for SARS-CoV-2 cell entry are weakly co-expressed in the placenta (49, 50). These findings could explain the rarity of placental SARS-CoV-2 infection and fetal transmission.

## 3. COVID-19 in the dermatology patient on IMBI therapy

There have been two essential questions in the medical community: a) whether IMBI therapy increases the risk of COVID-19 or subsequent mortality and b) whether IMBI should be postponed or discontinued in patients diagnosed with COVID-19. Regarding the first question, a retrospective matched cohort study from Massachusetts showed that, overall, biologics were not associated with COVID-19 (OR, 0.88; 95% confidence interval [CI], 0.71–1.09; *p*¼ 0.25), adjusting for demographics, comorbidity burden, and local infection rates (51). Patients treated with TNFαI were less likely to be diagnosed with SARS-CoV-2 infection than matched controls (OR, 0.69; 95% CI, 0.48–0.98; *p*¼0.04). Mortality rates were similar in the group of patients on biologics and matched controls. In another study, IMBI therapy was not associated with increased COVID-19 severity in patients with hidradenitis suppurativa (52). A retrospective chart review from the Houston greater area adds to the evidence that patients on biologics are not at increased risk of contracting COVID-19 or have worse outcomes, except for those on rituximab (shown to increase hospitalization rate) (53). Interestingly, the study’s results suggested that patients on biologics have a lower rate of COVID-19 positivity than the general population, suggesting a possible protective effect.

Most guidelines of professional bodies concur that discontinuation of treatment for the concern of contracting COVID-19 is not supported because it may lead to decreased efficacy outcomes with reintroduction or a flare of conditions such as psoriasis (54, 55). The guidance of the American Academy of Dermatology (AAD) and other medical societies do not recommend routine discontinuation of IMBI therapy in patients who have not tested positive for COVID-19 or exhibited signs/symptoms of the disease (55, 56). However, one should consider risk vs. benefit in each patient, considering the severity of the dermatologic condition and risk factors for severe manifestations of COVID-19 disease (including age > 60, cardiovascular disease, hypertension, and diabetes) (56, 57). Also, patients with dermatologic disorders, including psoriasis, hidradenitis suppurativa, and atopic dermatitis that are associated with metabolic syndrome, older age, or comorbidities such as respiratory disorder have poorer prognosis if they become infected with COVID-19 (58). A higher risk of all-cause pneumonia has been reported in patients with severe skin disorders such as psoriasis.

AAD and *National Institute for Health and Clinical Excellence (NICE) Guideline 169* for dermatologic conditions recommend the discontinuation of biologics in COVID-19-positive patients (55, 56, 59). However, when deciding whether to stop treatment, the healthcare provider should consider the severity of COVID-19, the risks and benefits of stopping treatment, the severity of the dermatologic condition, and the effect of withholding treatment on concomitant non-dermatologic conditions (59). Patient may re-initiate IMBI therapy after complete recovery from COVID-19. Some authors recommend continuing biologic, especially TNFαI medications that are known to suppress cytokine storms if viral symptoms are mild (60). A recent systematic review of professional bodies’ immunosuppressant guidelines during the COVID-19 pandemic stated that steroid usage should not be stopped abruptly (55). Also, it advised an individualized risk–benefit analysis considering the risk of the effect of COVID-19 infection, including the psychological burden, and the likelihood of relapse of the condition treated with IMBI.

The British Association of Dermatologists (BAD) recommends shielding for patients at the highest clinical risk: patients taking ⩾20 mg prednisolone (or equivalent) per day for >4 weeks or ⩾5 mg prednisolone (or equivalent) per day combined with another immunosuppressant, patients taking a combination of two or more immunosuppressants, patients taking cyclophosphamide, and patients on rituximab or infliximab for primarily skin conditions (61).

## 4. COVID-19 vaccination in the dermatology patient on IMBI therapy

Most professional associations concur that COVID vaccines are safe in the dermatology patient on IMBI (62–66). As none of the COVID-19 vaccines developed are live attenuated vaccines, they do not pose the risk of vaccine-induced infection, a significant concern in immunosuppressed patients. No data support that patients on IMBI exhibit a higher frequency of adverse effects from COVID-19 vaccines compared to persons not on IMBI (67). In patients with a history of anaphylaxis to vaccinations, systemic mastocytosis or idiopathic anaphylaxis, an allergy diagnostic work-up should be performed prior to vaccination (68, 69). Dermatologic complications of COVID-19 vaccines are typically mild and self-limited (70, 71).

A study suggested that there might be an association between COVID-19 vaccination and exacerbation of autoimmune bullous diseases (AIBDs) (72). Three-fourths of patients were on systemic therapy, mostly prednisolone (73.1%), at the time of vaccination. Patients vaccinated in the active phase of the disease were more likely to experience post-vaccine disease exacerbation, with a number needed to harm of 3. Still, the authors indicated that the benefits of vaccination outweigh the potential risk of complications. Vaccination is recommended for such patients, but preferably in the remission/controlled phase of the disease.

Although there are no direct supporting data, based on studies with other vaccines, there has been a concern that some immunomodulators, particularly if more than one is being used, may diminish COVID-19 vaccine efficacy (64). However, newer generation biologic agents used in chronic plaque psoriasis and atopic dermatitis showed little to no interference with seasonal influenza, pneumococcal, or tetanus vaccine (73–75). Variable humoral responses to hepatitis B (HBV) vaccine were reported in patients on IMBI (76, 77). Good antibody levels were observed after vaccination for patients on IL-17 (e.g., secukinumab) and IL-4/13 inhibitors (dupilumab) (62). TNF*α*I (adalimumab, certolizumab, etanercept) and anti-IL-12/23 (ustekinumab) biologics have been associated with a decrease in antibody levels. Prednisone at a dose of >20 mg per day diminished humoral responses to influenza vaccines in patients with systemic lupus erythematosus (78). While cyclosporin treatment results in severely disturbed humoral responses to vaccines in kidney transplant recipients, this may not be generalized to patients with dermatology immune disease (62).

An essential study investigated the effect of immunosuppression on the immunogenicity of mRNA COVID-19 vaccines in patients with chronic inflammatory disease (79). The authors noted that S-specific antibody titers observed in patients on TNFαI were similar to those in patients with rapid recovery from COVID-19 and may provide sufficient humoral protection. However, anti-S IgG antibody titers after vaccination were lower in participants receiving glucocorticoids than in those not. A US study indicated that mRNA vaccine efficacy against COVID-19-associated hospitalization was lower in patients with a rheumatologic or inflammatory disorder (81%) than in immunocompetent persons (90%) (80). The authors concluded that immunocompromised persons benefit from mRNA COVID-19 vaccination but are less protected from severe COVID-19 outcomes than immunocompetent persons.

A study by Simon and *colleagues* indicated that immune responses against the SARS-CoV-2 are delayed and reduced in patients with immune-mediated inflammatory diseases (81). This effect was attributed to the disease itself rather than concomitant treatment. A study of 50 patients with stable plaque psoriasis treated with biologics for at least 2 months examined mRNA vaccine safety (participants received 2 doses) and subsequent psoriasis flares (82). All patients discontinued their biological therapy 10 days before and 10 days after each vaccine dose. Of these, 24 patients were treated with TNFαI, 14 with anti-IL17, 7 with anti-IL12-23, and 5 with anti-IL23. All patients were evaluated on days 2, 7, and 14 post-vaccination for local and/or systemic side effects and/or reactions to the vaccine. None of the patients experienced adverse effects or a psoriatic flare. Only one patient treated with infliximab biosimilar referred an exacerbation of psoriasis after vaccination. These findings supported that SARS-CoV-2 mRNA vaccines are safe for patients with chronic plaque psoriasis treated with biologics and do not trigger psoriasis. However, these data should be validated in a larger sample.

A multidisciplinary committee provided guidance on the safety and efficacy of SARS-CoV-2 vaccination for dermatologists and other clinicians when prescribing IMBI therapies (62). It concluded that the SARS-CoV-2 vaccines approved are expected to be safe for dermatology patients on IMBI. There is variability in vaccine efficacy, depending on the degree of immunosuppression which in turn depends on the type of IMBI therapy, dose, duration, general condition of the patient, and the type of vaccine administered. Data generally support a possible decrease in antibody titers with TNFαI, rituximab, ustekinumab, and many oral immunotherapies, including corticosteroids. The risk–benefit ratio may favor immunization if immunosuppression is low and there is a significant risk of COVID-19 development. One may consider checking antibody titers after vaccination and using additional vaccinations, if needed, to boost protective antibodies. If protective antibody titers are inadequate and skewed to a T helper type 2 phenotype, vaccine-associated enhanced respiratory disease (VAERD) can develop (83).

Regarding the timing of COVID-19 vaccination, AAD and BAD do not recommend expediting vaccination before planning IMBI therapy for skin disease (62, 63). However, guidance from other authorities, such as the EADV and Australasian Medical Dermatology Group, recommends that vaccination be expedited and administered prior to initiation of IMBI (64, 65).

## 5. COVID-19 in the pregnant patient on IMBI therapy

There exists limited data surrounding the trajectory of COVID-19 in pregnant women on IMBI. A theoretical concern is that IMBI therapies causing moderate to severe immunosuppression may increase maternal/fetal risks from COVID-19 because of delayed clearing of the viral mRNA. However, there is hardly any supporting evidence in the literature. In one case study, a pregnant patient with severe ulcerative colitis and COVID-19 infection experienced a spontaneous abortion following treatment of COVID-19 with azithromycin and hydroxychloroquine and treatment of ulcerative colitis with intravenous cyclosporine (84). However, the cause of the spontaneous abortion was unclear. Several authors suggest that there is no need to abruptly discontinue IMBI therapy in pregnancy in patients with quiescent inflammatory bowel disease (IBD) (85).

Biologics such as TNFαI decrease cytokine storms and, therefore, may be associated with minimal risk in the pregnant patient and can be handled as in the non-pregnant person. There have been no safety signals in the literature regarding TNFαI use in pregnancy in the context of COVID-19. A retrospective study included 244 pregnant women with IBD, of which 75 (30.7%) were on biologics; in 22 of those (29.3%), the treatment was stopped at a median of 28 weeks’ gestation (85). Twenty-two (9%) of patients were on systemic corticosteroids. Despite high levels of immunosuppression, only a single COVID-19 infection occurred. Adverse pregnancy outcomes were infrequent and not associated with IMBI.

Antenatal systemic corticosteroids are administered to women at threat of preterm birth and fetal lung maturity and confer significant morbidity and mortality benefit for neonates (86). The RECOVERY trial recommends administering prednisolone 40 mg orally or intravenous hydrocortisone 80 mg twice daily in COVID-19 patients who require oxygen supplementation or ventilatory support (87). However, this trial included only six pregnant patients. Other authors recommended methylprednisolone because of its limited placental transfer and documented efficacy in cases of acute lung injury (88). Most authors concur that corticosteroid administration should continue in the context of iatrogenic preterm delivery in pregnant COVID-19 patients due to maternal condition (2). While these findings cannot be extrapolated to the use of corticosteroids for non-obstetric reasons, such as a severe flare of a dermatologic condition, they indicate that short courses of systemic steroids should not be excluded in the management of dermatologic conditions in pregnancy, and a risk–benefit analysis should be conducted, as in the non-pregnant patient. Controlling the dermatologic condition is often crucial to the successful completion of pregnancy and minimizing fetal risks associated with the condition. When using a systemic steroid, the provider should assess the therapeutic effect as soon as possible and try to taper the dose of systemic steroid rapidly.

## 6. COVID-19 vaccination in the pregnant woman on IMBI therapy

Most authorities recommend that all unvaccinated people planning a pregnancy or those who are pregnant or recently pregnant undergo COVID-19 vaccination, and those who are vaccinated should receive booster doses when eligible (36, 89, 90). The recommendation for vaccination during pregnancy is based on data showing vaccine safety and efficacy in pregnant people and data that pregnancy is associated with an increased risk of severe infection (36). Studies on vaccination among pregnant women (most participants vaccinated in the third trimester) show no evidence of harmful fetal/perinatal effects such as neonatal death, stillbirth, congenital anomalies, decreased fetal growth, preterm birth, or miscarriage (91–93). Several studies reported worse pregnancy outcomes in unvaccinated COVID-19-positive patients (94). In a US study, mRNA COVID-19 vaccination series during pregnancy was associated with reduced risk for COVID-19 hospitalization among infants (95). Vaccine efficacy against admission to an ICU for COVID-19 was 70 percent, 90 percent of the infants admitted to an ICU for COVID-19 were born to unvaccinated mothers, and the only two infants who died were born to unvaccinated mothers.

Experts suggest that women during pregnancy or postpartum should opt for mRNA vaccines, if accessible, as viral vector vaccines may cause thrombosis associated with thrombocytopenia (91). If mRNA vaccines are unavailable, any viral vector vaccine is deemed better than no vaccine (36). Regarding the timing of a pregnancy after undergoing the first or both COVID-19 vaccine doses, experts maintain that there is no impact on pregnancy and that vaccination against SARS-CoV-2 infection should occur or continue based on standard protocols (36, 96).

There are inadequate data on vaccine safety and efficacy in pregnant patients on IMBI. In a descriptive study, unvaccinated pregnant women with rheumatic and musculoskeletal diseases (RMD) and COVID-19 had a greater number of pre-term births compared with those fully vaccinated against COVID-19 (97). Additionally, the need for COVID-19 pharmacological treatment was uncommon in pregnant women with RMD regardless of vaccination status. These results support active promotion of COVID-19 vaccination in women with RMD who are pregnant or planning a pregnancy. Of note, a study of 113 patients with rheumatic disease (68% of the patients were on IBMI and 2% pregnant) did not show any association between the medications and COVID-19 mortality (except for rituximab); mortality in this cohort was associated with comorbidities, especially diabetes, obesity, and interstitial lung diseases (98).

In a report of two pregnant COVID-19-positive systemic lupus erythematosus patients, both women delivered healthy babies (99). One patient presenting at 38 weeks of gestation did not discontinue or decrease the use of azathioprine, hydroxychloroquine, and prednisone but was induced on the same day as her positive COVID-19 test. The other patient, who was taking hydroxychloroquine, azathioprine, and etanercept, tested positive for COVID-19 at 19 weeks of gestation and discontinued azathioprine and etanercept, but resumed both soon after. Etanercept was stopped at 30 weeks of gestation. She later received triamcinolone acetonide injections for oligoarthritis. She underwent cesarean section complicated by a placenta accrete resulting in a massive hemorrhage; however, the patient and newborn were both discharged in good health 3 days after delivery.

No data support that the COVID-19 vaccine response in a pregnant patient on IMBI differs from that in a non-pregnant counterpart. There is evidence that vaccination of a non-pregnant patient on IMBI provokes substantial humoral responses, even if the antibody titers generated are lower in patients on certain medications such as systemic corticosteroids. Most importantly, looking at the rheumatic disease group of patients, which overlaps significantly with the dermatologic disease group, IMBI in a non-pregnant patient was not associated with COVID-19 mortality (except for rituximab), and vaccination of the rheumatic patient during pregnancy improved the obstetric outcomes compared to the unvaccinated patient. Based on this data, it can be stated that after weighing the benefit–risk profile of the available COVID-19 vaccines, the recommendation for the pregnant dermatology patient speaks in favor of the COVID-19 vaccination.

## 7. Counseling the pregnant patient on IMBI therapy

Several issues should be included in a consultation about the risks and benefits of discontinuing IMBI therapy:

- Scarcity of data on pregnancy during COVID-19 clinical trials
- Studies showing that IMBI may not affect the course of COVID-19 in immunocompetent patients with dermatologic conditions (51–53)
- Data showing that adverse pregnancy outcomes were infrequent and not associated with IMBI in IBD patients (85)
- Risk of COVID-19 complications due to pregnancy (increased risk to a pregnant person of severe disease and death) or underlying conditions (e.g., diabetes, obesity, heart disease)
- Risk of COVID-19 to the fetus or newborn (preterm birth rate appears to be increased)

The above issues should also be discussed in a consultation about COVID-19 vaccination during pregnancy (100). Additionally, the healthcare provider should discuss the following:

- Risk of vaccine reactogenicity, including fever (treatment with antipyretic medications such as acetaminophen decreases this risk) and dermatologic adverse effects (self-limited) (70)
- Time of planned vaccination during pregnancy (fever in the first trimester caused by the vaccine may increase the risk of congenital defects)
- Extensive evidence for safety of other vaccines during pregnancy
- Studies favoring vaccination in pregnancy in a similar group, the rheumatology patients (97, 98)
- A potential protective effect for the neonate from the placental transfer of antibodies to the fetus (101)
- Risk of exposure to SARS-CoV-2 and potential for mitigation with working from home, wearing masks, and physical distancing.

The above maternal and fetal risks need to be weighed against the risks of COVID-19 itself.

The pregnant individual should be advised that there are no biologic reasons to believe that the vaccines currently approved are harmful to the pregnant person or fetus (102, 103). A case of normal delivery in a pregnant female vaccinated for COVID-19 during third trimester showed the presence of antibodies in the baby indicating a possible protective effect for the neonate (101, 104). The available evidence, theoretical considerations, FDA evaluation, Centers of Disease Control (CDC), and professional medical society guidance point in the same direction: the real benefits of COVID-19 immunization in the immunocompetent pregnant individual outweigh the scant theoretical safety concerns in pregnancy. The risk of having a lowered response to COVID-19 vaccine because of concomitant IMBI therapy taking exists, but a lowered response is better than lack of protection (vaccine not administered). This should be discussed in the consultation. A shared decision-making among the mother, her family, and health care provider is warranted pending further data from clinical trials and vaccinated pregnant persons outside clinical trials (100, 103).

## 8. Conclusion

There is insufficient evidence regarding the continuation of IMBI therapy for dermatologic conditions in pregnant persons diagnosed with COVID-19. However, available data indicate that there is no compelling reason for treating pregnant patients differently than non-pregnant. The risks of COVID-19 vaccination in pregnant patients on IMBI therapy for dermatologic conditions can be extrapolated from experimental studies and studies in overlapping populations such as rheumatology patients. Current guidelines on COVID-19 vaccination for immunosuppressed dermatologic patients and pregnant patients separately recommend vaccination in both groups. The body of evidence indicates that mRNA COVID-19 vaccines are safe during pregnancy. Along the same lines, many biologics can be safely administered during COVID-19–some TNFαI can even benefit the course of the disease. Based on the evidence presented here, COVID-19 vaccine recommendations in pregnant patients on IBMI for dermatologic conditions should not differ from those for their non-pregnant counterparts. Collecting data for post-vaccine surveillance will help address some of the unanswered questions.

## Author contributions

All authors listed have made a substantial, direct, and intellectual contribution to the work and approved it for publication.

## Conflict of interest

The authors declare that the research was conducted in the absence of any commercial or financial relationships that could be construed as a potential conflict of interest.

## Publisher’s note

All claims expressed in this article are solely those of the authors and do not necessarily represent those of their affiliated organizations, or those of the publisher, the editors and the reviewers. Any product that may be evaluated in this article, or claim that may be made by its manufacturer, is not guaranteed or endorsed by the publisher.

## Supplementary material

The Supplementary material for this article can be found online at: https://www.frontiersin.org/articles/10.3389/fmed.2023.1121025/full#supplementary-material
