# Supplementary material for: A critical review of COVID-19 course and vaccination in dermatology patients on immunomodulatory/biologic therapy: recommendations should not differ between non-pregnant and pregnant individuals
Source: Front Med (Lausanne). 2023 Jun 2;10:1121025. doi: 10.3389/fmed.2023.1121025 (PMC10272467; doi:10.3389/fmed.2023.1121025)
Supplement: Supplementary file 1 [file Table_1.DOC]

| **Table 1. Safety of systemic immunomodulatory therapy in pregnancy.** | | | | |
| --- | --- | --- | --- | --- |
| **Medication** | **PLLR labeling*** | | | **Comments and Recommendations†** |
|  | **Animal and Human Studies** | **Pregnancy Registry** | **Risk Summary and Clinical Considerations** |  |
| **Antimalarials** (chloroquine phosphate, hydroxychloroquine phosphate [HCQ]) | *Animals:* no studies with HCQ; chloroquine studies show teratogenicity at supratherapeutic doses and accumulation in fetal ocular melanin structures  *Humans:* no teratogenicity; large clinical series in malaria and SLE are reassuring | Yes (HCQ) | Studies in pregnant women have not identified a drug-associated risk of MBDs, miscarriage, or adverse maternal/fetal outcomes | Risk of SLE flare greater than risk of drug AEs; therefore, continue HCQ during pregnancy |
| **Cyclosporine** | *Animals:* nephrotoxicity, VSDs with supratherapeutic dose  *Humans:* LBW and prematurity, 27% neonatal complications in 116 pregnancies (90% transplant patients); unclear whether these AEs relate to medication or underlying disease; reports of malformation with inconsistent pattern | Yes | No well-controlled studies in pregnant women; the risk/benefit ratio of using the drug in psoriasis patients during pregnancy should carefully be weighed with serious consideration for discontinuation | Drug may be used for the shortest duration possible if benefit outweighs maternal/fetal risks; wide spectrum of drug interactions |
| **IVIg** | *Animals:* no studies  *Humans:* no studies, no reported AEs | No | Drug-associated risk is unknown because of lack of safety data in humans | Used in treatment of pemphigoid gestationis, pregnancy-related autoimmune conditions, rubella post-exposure prophylaxis |
| **Methylprednisolone,**  **Prednisolone** | *Animals*: oral cleft, placental insufficiency, spont abortion, IUGR  *Humans:* oral cleft, LBW | No | Drug should not be used during pregnancy unless the potential benefit justifies the potential risk to the mother/fetus | Converted by placental enzymes to inactive prednisone or less active cortisone, thus only 10% of maternal dose reaches fetus; risks related to daily dose, but most importantly, duration of Rx; short courses can be considered in 2^nd^ and 3^rd^ trimesters; fetal risks include HPA suppression (important if used near time of delivery), immunosuppression, congenital cataract |
| **Prednisone** | *Animals:* oral cleft, placental insufficiency, spont abortion, IUGR  *Humans:* oral cleft, LBW, prematurity, abortion, congenital cataract | No | Drug should not be used during pregnancy unless the potential benefit justifies the potential risk to the mother/fetus | Short courses can be considered in 2^nd^ and 3^rd^ trimesters; risks related to daily dose, but most importantly, duration of Rx; no ↑ risk of congenital anomalies with doses 40-80 mg/d for short periods but no controlled data; fetal risks include HPA suppression (important if used near time of delivery), immunosuppression, congenital cataract |

Abbreviations: AEs, adverse effects; HCQ, hydroxychloroquine; HPA, hypothalamus-pituitary axis; IUGR, intrauterine growth restriction; IVIg, intravenous immune globulin; LBW, low birth weight; MBDs, major birth defects; Rx, treatment; SLE, systemic lupus erythematosus; VSD, ventricular septal defect

*Information available at <https://dailymed.nlm.nih.gov/dailymed/drugInfo.cfm>

**†**Mutyambizi K, Mackool BT. Drug safety. In: Kroumpouzos G ed*. Text Atlas of Obstetric Dermatology*, Lippincott Williams & Wilkins, Philadelphia, 2013, pp. 217-250.
