# Supplementary material for: A critical review of COVID-19 course and vaccination in dermatology patients on immunomodulatory/biologic therapy: recommendations should not differ between non-pregnant and pregnant individuals
Source: Front Med (Lausanne). 2023 Jun 2;10:1121025. doi: 10.3389/fmed.2023.1121025 (PMC10272467; doi:10.3389/fmed.2023.1121025)
Supplement: Supplementary file 2 [file Table_2.DOC]

| **Table 2. Safety of systemic biologic therapy in pregnancy.** | | | | |
| --- | --- | --- | --- | --- |
| **Medication** | **PLLR labeling*** | | | **Comments and Recommendations**** |
|  | **Animal and Human Studies** | **Pregnancy Registry** | **Risk Summary and Clinical Considerations** |  |
| **Adalimumab** | *Animals:* no evidence of fetal harm  *Humans:* registry cannot reliably establish whether there is an a/w MBDs | Yes | Studies do not reliably establish a/w MBDs; lack of pattern of MBDs in registry; drug is actively transferred across the placenta during the 3^rd^ trimester and may affect immune response in the in-utero exposed infant | Low risk of teratogenic, embryotoxic, or fetotoxic effects; manufacturer rec use in pregnancy only when clearly needed |
| **Certolizumab pegol** | *Animals:* no evidence of fetal harm  *Humans:* small number of exposed pregnancies with known outcomes | Yes | Pregnancy registry data are insufficient to inform a risk of MBDs or other AEs; studies of the drug’s plasma concentrations during the 3^rd^ trimester showed that placental transfer was negligible or low in infants at birth | Low risk of teratogenic, embryotoxic, or fetotoxic effects; no late active placental transfer due to its unique structure without the Fc portion; may use in pregnancy only if the benefit outweighs the risk to the fetus |
| **Dupilumab** | *Animals:* no malformations, developmental or embryo-fetal toxicity  *Humans:* no studies | Yes | Case reports/series have not identified a drug-associated risk of MBDs, miscarriage, or other adverse maternal/fetal outcomes; human IgG antibodies cross the placental barrier; therefore, the drug may be transmitted from the mother to the fetus; there are adverse outcomes a/w asthma in pregnancy (preeclampsia in the mother, and prematurity, LBW, and small for gestational age in the neonate) | The level of asthma control should be closely monitored in pregnant women and Rx adjusted as necessary to maintain optimal control. |
| **Etanercept** | *Animals:* no evidence of fetal harm  *Humans:* no well-controlled studies; reports of anomalies that are part of VACTERL association have not been confirmed; no other reports of congenital anomalies | Yes | No MBDs in studies; lack of pattern of major birth defects in registry; the risk of fetal/neonatal adverse reactions with in-utero exposure to the drug is unknown | Low risk of teratogenic, embryotoxic, or fetotoxic effects; manufacturer rec use in pregnancy only when clearly needed |
| **Infliximab** | *Animals:* no developmental AEs with anti-TNF analogous antibody  *Humans:* 2 prospective cohorts showed no malformations or other AEs; reports of anomalies that are part of VACTERL association have not been confirmed | Yes | No increased risk of MBDs in women exposed to infliximab as compared to those exposed to non-biologics; drug detected in the serum of infants up to 6 months following birth; therefore, these infants may be at increased risk of infection | Reaches maximal transplacental transport capacity in 3^rd^ trimester, with levels up to 3x maternal serum levels; some authors rec stop at week 30 and resume after delivery |
| **Secukinumab** | *Animals:* no malformations or embryo-fetal toxicity  *Humans:* no safety signals regarding spont abortions and malformations were identified in manufacturer’s database | No | Risk is unknown because of limited available human data; reassuring animal data | As an IgG1 molecule, secukinumab could theoretically cross the placenta, but most antibody transfer occurs in the 3^rd^ trimester; may use during pregnancy only when clearly needed |
| **Ustekinemab** | *Animals:* no malformations or developmental toxicity  *Humans:* no well-controlled studies | No | Risk is unknown because of limited available human data; reassuring animal data | Manufacturer rec use in pregnancy only if benefit justifies fetal risk |

Abbreviations: AEs, adverse effects; a/w, associated/association with; MBDs, major birth defects; rec, recommends; Rx, treatment; VACTERL, Vertebral defect Anal atresia Cardiac defect Tracheo-Esophageal, Renal and Limb abnormalities

*Information available at <https://dailymed.nlm.nih.gov/dailymed/drugInfo.cfm>

**†**Mutyambizi K, Mackool BT. Drug safety. In: Kroumpouzos G ed*. Text Atlas of Obstetric Dermatology*, Lippincott Williams & Wilkins, Philadelphia, 2013, pp. 217-250.
